# Protective Effects of Danhong Injection against Cerebral Damage during On-Pump Coronary Artery Bypass Graft Surgery

**DOI:** 10.1155/2015/527219

**Published:** 2015-12-22

**Authors:** Zhang Xuejuan, Zhang Jietao, Han Di, Zheng Yu, Guo Xiaozi, Li Yunfa, Dong Lihua

**Affiliations:** ^1^General Medicine, Affiliated Hospital of Qingdao University, Qingdao 266031, China; ^2^Department of Neurology, Rizhao People's Hospital, Rizhao 276826, China

## Abstract

To explore the protective effects of Danhong injection against cerebral damage during on-pump coronary artery bypass graft surgery and its mechanism.* Methods*. Fifty patients scheduled for on-pump CABG surgery were randomly divided into Danhong injection group (group D) and control group (group C). Group D was given Danhong injection while group C was given the same volume of normal saline when the artery was cut open. Jugular bulb blood right before the operation began (T1), when body temperature rewarming to 36°C (T2), 30 min after the termination of cardiopulmonary bypass (T3), and 6 hrs after the termination of CPB (T4) was collected. The superoxide dismutase activity by using xanthine oxidase method and concentration determination of malondialdehyde were examined.* Results*. In group C, SOD activity was less at T2–T4 than at T1. It was also less active comparatively in group D at T2–T4. The MDA concentration increased in both groups but was more obvious in group C. Levels of TNF-*α*, IL-6, IL-8, and IL-10 increased in both groups C and D at T3 and T4, compared to T1.* Conclusions*. Danhong injection shows significant protective effects against cerebral damage during on-pump coronary artery bypass graft surgery.

## 1. Introduction

The protection of vital organs against damage during cardiopulmonary bypass (CPB) cardiac surgery is a must thing for any health professional. It has been reported that there is damage induced by ischemia reperfusion during on-pump coronary artery bypass graft surgery [1]. This has aroused increasing interest among researchers and has given new direction to the extensive and in-depth study on how to protect heart, liver, kidney, and so forth during CPB. However, very few researches have been done on cerebral damage during this procedure. Danhong injection mainly consists of Chinese herbs Danshen and Honghua. It is often used for treating coronary heart disease, angina, hypertension, cerebral thrombosis, and other cardiovascular diseases clinically. The protective effects of Danhong injection against cerebral damage during on-pump coronary artery bypass graft surgery have not been reported yet. In this study, we focus on the effects of anti-inflammation, antioxidation, and regulating immune factors induced by Danshen injection in the patients who undergo on-pump coronary artery bypass graft surgery. Our aim is to evaluate the protective effects of Danhong injection against cerebral damage during this surgery and its potential mechanism.

## 2. Materials and Methods

### 2.1. Subjects Selection

Fifty patients aged 50–75 years, who met the following criteria, were recruited for on-pump coronary artery bypass grafting. The criteria are no histories of endocarditis, nervous system disease, immune system disease, or mental disorder; absence of abnormal function of lung, liver, or kidney; presentation of normal hemoglobin level, electrolyte concentration, and blood gas analysis results; presentation of cardiac function of grades II-III (categorization according to the New York Heart Association Functional Classification); and no use of drugs that may affect the central nerve system. These eligible subjects were randomly divided into Danhong injection group (group D) and control group (group C) with 25 patients in each group.

### 2.2. Operation Procedures

Patients fasted for 12 hrs prior to the operation and their fluid intake was forbidden for 4 h. Anesthesia was induced with intramuscular administration of sodium (0.1 g) and scopolamine (0.3 mg) and intravenous administration of midazolam (0.1 mg·kg^−1^), fentanyl (10–20 *μ*g·kg^−1^), and vecuronium bromide (0.1–0.2 mg·kg^−1^). After intubation, mechanical ventilation was used to maintain normal arterial blood carbon dioxide tension. Then radial artery and right internal jugular vein catheterization was performed; particularly, jugular vein catheterization was retrograded until it reached the bulb of jugular vein (the inserted end of the catheter was placed parallel to the external auditory canal level). Additional administration of midazolam, fentanyl, and vecuronium was used intermittently to maintain anesthesia during the operation. After heparinization, CPB was performed using Sarns 9000 heart-lung machine and membrane oxygenator was used to maintain nonpulsatile perfusion rate of 60–80 mL·kg^−1^·min^−1^ with a microthrombi filter being placed in the arterial end of the machine. Average arterial pressure was maintained at 50–80 mmHg, and body temperature cooled until nasopharyngeal temperature was 29–33°C. Besides, the rewarming process should be slowed down to 1°C every 4-5 min. 10–15 minutes after the aorta was cut open, appropriate dose of dopamine was used to maintain circulation; during the CPB, pH value was kept at *α*-stat; no epinephrine was used during the observation period.

### 2.3. Drug Intervention

To evaluate the effects of Danhong injection, group D was given Danhong injection at the dose of 1.5 mL·kg^−1^, while group C was given the same volume of normal saline when the artery was cut open.

### 2.4. Blood Collection

Jugular bulb blood of each patient was collected at four time points including the following: right before the operation began (T1), when body temperature rewarming to 36°C (T2), 30 min after the termination of cardiopulmonary bypass (CPB) (T3), and 6 h after the termination of CPB (T4). The collected blood was used for the evaluation of superoxide dismutase (SOD) activity by xanthine oxidase method, concentration determination of malondialdehyde (MDA) by thiobarbital method, detection of TNF-*α*, IL-6, and IL-8 levels with radioimmunoassay, and determination of IL-10 concentration with ELISA.

### 2.5. Data Analysis

Data were analyzed using SPSS-11.0 statistical software and measurement data were presented as mean ± standard deviation. Comparison between both groups was treated with two-factor analysis of variance, and *P* < 0.05 was valued as statistically significant difference.

## 3. Result

No significant difference regarding age, gender, weight, length of CPB time, aortic clamp time, and operative time or blood gas analysis results between the two groups was found (*P* > 0.05, Table 1).

MDA concentration and SOD activity comparison was shown in Table 2. In group C, SOD was less active at T2 compared with that at T1; it was also less active compared with that in group C at T1 and T4. A rational application of SOD gene therapy is helpful for protection of the impairment of autoregulatory cerebral blood flow during the acute stage of subarachnoid hemorrhage. MDA concentration increased in both groups but was more obvious in group C. Evidence of increased prooxidant level associated with decreased protective, antioxidative factors suggests their mutual involvement in this complex pathology. So the Danhong injection can increase SOD and decrease MDA greatly with the acute cerebrovascular accident.

Comparison of TNF-*α*, IL-6, IL-8, and IL-10 levels between the two groups is shown in Table 3. TNF-*α*, IL-6, IL-8, and IL-10 levels in both groups are increased comparatively at T3 and T4 compared to T1. This increment was more obvious in group C with exception of IL-10 which increased more in group D at T4.

The correlation of SOD, MDA, TNF-*α*, IL-6, IL-8, and IL-10 to each other is shown in Table 4. From Table 4, the correlation coefficients are lower than 0.4, so there is no collinearity between the two indices.

## 4. Discussion

It has been reported that cerebral damage during CPB surgery is related to inflammatory response [2]. Then it is possible to alleviate the damage by restraining the inflammatory response during the CPB surgery procedure, which is supported by Murkin and Taylor, who have pointed out that the inhibition of inflammatory response is an important way to reduce the cerebral damage caused by CPB [3].

During inflammatory processes, endothelial cells become activated and expression of adhesion molecules is increased in response to inflammatory cytokines leading to aggregation of large number of white blood cells, which can contribute to cerebral microcirculatory disturbance through mechanical obstruction and vascular dilatation, resulting in cerebral hypoxia and ischemia. On the other hand, white blood cells aggregation can also induce damage to brain cells and blood brain barrier by releasing toxins like free oxygen radicals, proteolytic enzymes, lysosomal enzymes, and excitatory amino acid [3–5]. Furthermore, free oxygen radicals can stimulate the release of more cytokines, which in turn aggravate the damage. Chen et al. pointed out that the inhibition of inflammatory response is an important way to reduce the cerebral damage caused by CPB [6].

Danhong injection mainly consists of Chinese herbs Danshen and Honghua. It is used for treating coronary heart disease, angina, hypertension, thrombosis, and other cardiovascular diseases and has significant therapeutic effects on acute cerebral infarction [7]. Inflammation response plays an important role in the occurrence and development of atherosclerosis [8]. Relevant experiment showed that Danhong injection can significantly inhibit atherosclerosis in animal models, which is mainly related to its anti-inflammation and antioxidation functions. It was also shown that pretreatment of Danhong injection can protect endothelial cells from oxidative damage.

Till now, most studies on Danshen were focused on its function of lipid peroxidation, endothelial cell protection, and regulation of lipid and smooth muscle cells, while relevant researches about its effects on inhibiting infiltration of inflammatory cells and expression of immune factors were few. In this study, protective effects of SOD are enzymes that alternately catalyze the dismutation (or partitioning) of the superoxide (O_2_^−^) radical into either ordinary molecular oxygen (O_2_) or hydrogen peroxide (H_2_O_2_). Superoxide is produced as a byproduct of oxygen metabolism and, if not regulated, causes many types of cell damage. Hydrogen peroxide is also damaging, but less so, and is degraded by other enzymes such as catalase. Thus, SOD is an important antioxidant defense in nearly all living cells exposed to oxygen. Reducing glutathione can directly scavenge free radicals and protects biomolecules from their attack, and the GPx is required to characterize the changes in overall spectrum of the antioxidative stress system which may be relevant in providing defense against ROS exaggerated due to hypoxia in brain. MDA is a reactive aldehyde and is one of the many reactive electrophile species that cause toxic stress in cells and form covalent protein adducts referred to as advanced lipoxidation end-products. The production of this aldehyde is used as a biomarker to measure the level of oxidative stress in an organism. Danhong injection against cerebral damage during on-pump coronary artery bypass graft surgery was observed and it was shown that SOD was more active in group D after administration of Danhong injection, while MDA concentration was lower comparing with that in the control group (group C). TNF alpha is a cytokine that has a wide variety of functions. It can cause cytolysis of certain tumor cell lines; it is involved in the induction of cachexia; it is a potent pyrogen, causing fever by direct action or by stimulation of interleukin-1 secretion; and it can stimulate cell proliferation and induce cell differentiation. IL-6 plays an essential role in the final differentiation of B cells into IG-secreting cells, as well as inducing myeloma/plasmacytoma growth, nerve cell differentiation, and, in hepatocytes, acute-phase reactants. IL-10 is a protein that inhibits the synthesis of a number of cytokines, including IFN-gamma, IL-2, IL-3, TNF, and GM-CSF produced by activated macrophages and by helper T cells.

IL-8 may induce NGF production in vivo after brain injury. This hypothesis is also supported by the findings that astrocytes and microglia express the IL-8 receptor, and therefore these cells are likely candidates for the production of NGF following the challenge with IL-8. In addition, IL-8 has been shown to promote the survival of hippocampal neurons possibly through the release of factors by astrocytes that were present in these cultures. It cannot be excluded for the release of these factors. Therefore, to elucidate whether IL-8 induces NGF production in astrocytes, CSF samples of brain-injured patients containing IL-8 were incubated with purified astrocyte cultures derived from newborn mouse brain. NGF production was induced by all samples, except for one containing low levels of IL-8, and the NGF levels appeared to depend on the concentration of IL-8. When CSF samples were pretreated with anti-IL-8 antibodies, a distinct but not complete inhibition of the astrocytic NGF production occurred, indicating that IL-8 may act in association with other factors/cytokines. For this purpose, the CSF samples utilized for the culture experiments were tested for other cytokines. IL-6 and TNF-*α* were found. In both groups, concentration of TNF-*α*, IL-6, IL-8, and IL-10 increased at T3-4, comparing with that at T1, but it was more obvious in group C except that IL-10 level increased more at T4 in group D. These results indicate Danhong injection can alleviate cerebral damage during CPB. This might be related to its function of inhibiting the release of proinflammatory cytokines, promoting anti-inflammatory cytokines release, inhibiting free radicals production, and maintaining SOD activity during CPB.

Chinese medicinal Danhon injection includes multiple chemicals. In the future, our work is to find the specified compound mechanism of the cerebral damage which is important.

## Figures and Tables

**Table 1:** The clinical and demographic characteristics of patients.

Parameter	Group D	Group C
Age	60.2 ± 3.6	59.7 ± 2.8
Gender (male/female)	1 : 1.05	1 : 1.09
Weight	62.2 ± 5.9	61.8 ± 4.6
Length of CPB time	71.7 ± 5.6	72.1 ± 6.3
Aortic clamp time	33.5 ± 2.0	32.1 ± 1.9

**Table 2:** MDA concentration and SOD activity comparison at four time points (*n* = 25, *x* ± *s*).

Parameter	Group	T1	T2	T3	T4
SOD (NU/mL)	C	80.2 ± 5.6	71.9 ± 3.1^*∗*^	70.4 ± 4.2^*∗*^	69.8 ± 3.3^*∗*^
	D	81.9 ± 4.8	80.2 ± 6.7^ΔΔ^	81.6 ± 4.5^ΔΔ^	74.8 ± 4.9^Δ^

MDA (mol/L)	C	4.69 ± 0.62	6.98 ± 0.37^*∗*^	7.26 ± 0.28^*∗*^	6.62 ± 0.41^*∗*^
	D	4.76 ± 0.48	6.02 ± 0.46^*∗*Δ^	6.51 ± 0.44^*∗*Δ^	6.13 ± 0.28^*∗*Δ^

Comparing with T1 within group C, ^*∗*^ *P* < 0.01; comparing between group D and group C, ^Δ^ *P* < 0.05, ^ΔΔ^ *P* < 0.01.

**Table 3:** Comparison of TNF-*α*, IL-6, IL-8, and IL-10 levels between the two groups at four time points (*n* = 25, *x* ± *s*).

Parameter	Group	T1	T2	T3	T4
TNF-*α* (ng/mL)	C	20 ± 4	24 ± 6	39 ± 5^*∗*^	32 ± 3^*∗*^
	D	19 ± 5	22 ± 4	30 ± 4^*∗*ΔΔ^	25 ± 6^*∗*Δ^

IL-6 (pg/mL)	C	91 ± 18	108 ± 26	230 ± 25^*∗*^	220 ± 28^*∗*^
	D	84 ± 12	95 ± 21	200 ± 17^*∗*ΔΔ^	171 ± 29^*∗*ΔΔ^

IL-8 (ng/mL)	C	0.99 ± 0.24	1.12 ± 0.25	2.38 ± 0.17^*∗*^	2.61 ± 0.54^*∗*^
	D	0.95 ± 0.29	1.06 ± 0.27	2.09 ± 0.22^*∗*ΔΔ^	2.23 ± 0.17^*∗*Δ^

IL-10 (ng/mL)	C	27 ± 4	59 ± 11	230 ± 31^*∗*^	133 ± 26^*∗*^
	D	26 ± 3	47 ± 16	238 ± 56^*∗*^	172 ± 32^*∗*ΔΔ^

Comparing with T1 within group C, ^*∗*^ *P* < 0.01; comparing between group D and group C, ^Δ^ *P* < 0.05, ^ΔΔ^ *P* < 0.01.

**Table 4:** The correlation of SOD, MDA, TNF-*α*, IL-6, IL-8, and IL-10.

	SOD	MDA	TNF-*α*	IL-6	IL-8	IL-10
SOD	1	0.25	0.30	0.19	0.41	0.07
MDA		1	0.13	0.17	0.10	0.21
TNF-*α*			1	0.25	0.38	0.09
IL-6				1	0.19	0.12
IL-8					1	0.36
IL-10						1

## References

[1] Murkin J. M. (1999). Etiology and incidence of brain dysfunction after cardiac surgery. *Journal of Cardiothoracic and Vascular Anesthesia*.

[2] Tseng E. E., Brock M. V., Kwon C. C. (1999). Increased intracerebral excitatory amino acids and nitric oxide after hypothermic circulatory arrest. *Annals of Thoracic Surgery*.

[3] Jiang B., Ren L. S., Zhang S. Y., Wang P., Chu K. Q. (2015). Clinical value of homocysteine, cystatin C and FFA levels in patients with ischemic cerebrovascular disease. *Cancer Cell Research*.

[4] Yano T., Anraku S., Nakayama R., Ushijima K. (2003). Neuroprotective effect of urinary trypsin inhibitor against focal cerebral ischemia-reperfusion injury in rats. *Anesthesiology*.

[5] Blaszczyk J., Kedziora J., Zaslonka J. (2000). Moderate systemic hypothermia and cold crystalloid cardioplegia influence on myocardial ischemic and revascularisative injury. *Medical Science Monitor*.

[6] Chen T.-T., Jiandong-Liu, Wang G., Jiang S.-L., Li L.-B., Gao C.-Q. (2013). Combined treatment of ulinastatin and tranexamic acid provides beneficial effects by inhibiting inflammatory and fibrinolytic response in patients undergoing heart valve replacement surgery. *Heart Surgery Forum*.

[7] Morita K. (2012). Surgical reoxygenation injury of the myocardium in cyanotic patients: clinical relevance and therapeutic strategies by normoxic management during cardiopulmonary bypass. *General Thoracic and Cardiovascular Surgery*.

[8] Komori M., Takada K., Tomizawa Y., Uezono S., Ozaki M. (2003). Urinary trypsin inhibitor improves peripheral microcirculation and bronchospasm associated with systemic anaphylaxis in rabbits in vivo. *Shock*.

